# Short-Course of Methylprednisolone Improves Respiratory Functional Parameters After 120 Days in Hospitalized COVID-19 Patients (Metcovid Trial): A Randomized Clinical Trial

**DOI:** 10.3389/fmed.2021.758405

**Published:** 2021-11-30

**Authors:** Camila Miriam Suemi Sato Barros, Raissa Soares Freire, Elisângela Frota, Anna Gabriela Rezende Santos, Maria Eduarda Leão Farias, Maria Gabriela Almeida Rodrigues, Bernardo Maia Silva, Christiane Maria Prado Jeronimo, Rebeca Linhares Abreu Netto, Mayla Gabriela Silva Borba, Djane Baía-da-Silva, José Diego Brito-Sousa, Mariana Simão Xavier, Marcia Almeida Araújo-Alexandre, Vanderson Souza Sampaio, Gisely Cardoso Melo, Guilherme Tinoco Arêas, Ludhmila Abrahão Hajjar, Wuelton Marcelo Monteiro, Felipe Gomes Naveca, Fábio Trindade Maranhão Costa, Fernando Fonseca Almeida Val, Marcus Vinícius Guimarães Lacerda, The Metcovid team, Adria de Lemos Vasconcelos

**Affiliations:** ^1^Instituto de Pesquisa Clínica Carlos Borborema, Fundação de Medicina Tropical Dr Heitor Vieira Dourado, Manaus, Brazil; ^2^Programa de Pós-Graduação em Medicina Tropical, Universidade do Estado do Amazonas, Manaus, Brazil; ^3^Programa de pós-Graduação em Ciências da Saúde, Universidade Federal do Amazonas, Manaus, Brazil; ^4^Instituto Leônidas & Maria Deane, Manaus, Brazil; ^5^Instituto Nacional de Infectologia Evandro Chagas, Rio de Janeiro, Brazil; ^6^Fundação de Vigilância em Saúde do Amazonas, Manaus, Brazil; ^7^Universidade Federal do Amazonas, Manaus, Brazil; ^8^Faculdade de Medicina, Universidade de São Paulo, São Paulo, Brazil; ^9^Universidade Estadual de Campinas, São Paulo, Brazil

**Keywords:** SARS-CoV-2, COVID-19, pulmonary function test, long-covid, corticosteroid, inflammation, coronavirus, Brazil

## Abstract

**Background:** The use of corticosteroids may help control the cytokine storm occurring in acute respiratory failure due to the severe form of COVID-19. We evaluated the postacute effect of corticosteroids used during the acute phase, such as impairment in pulmonary function parameters, on day 120 (D120)-follow-up, in participants who survived over 28 days.

**Methods:** This is a parallel, double-blind, randomized, placebo-controlled phase IIb clinical trial carried out between April 18 and October 9, 2020, conducted in hospitalized patients with clinical–radiological suspicion of COVID-19, aged 18 years or older, with SpO_2_ ≤ 94% on room air or requiring supplementary oxygen, or under invasive mechanical ventilation (IMV) in a referral center in Manaus, Western Brazilian Amazon. Intravenous methylprednisolone (MP) (0.5 mg/kg) was given two times daily for 5 days to these patients. The primary outcome used for this study was pulmonary function testing at day 120 follow-up visit.

**Results:** Out of the total of surviving patients at day 28 (*n* = 246) from the Metcovid study, a total of 118 underwent satisfactory pulmonary function testing (62 in the placebo arm and 56 in the MP arm). The supportive treatment was similar between the placebo and MP groups (seven [11%] vs. four [7%]; *P* = 0.45). At hospital admission, IL-6 levels were higher in the MP group (*P* < 0.01). Also, the need for ICU (*P* = 0.06), need for IMV (*P* = 0.07), and creatine kinase (*P* = 0.05) on admission also tended to be higher in this group. In the univariate analysis, forced expiratory volume on 1st second of exhalation (FEV1) and forced vital capacity (FVC) at D120 follow-up were significantly higher in patients in the MP arm, being this last parameter also significantly higher in the multivariate analysis independently of IMV and IL-6 levels on admission.

**Conclusion:** The use of steroids for at least 5 days in severe COVID-19 was associated with a higher FVC, which suggests that hospitalized COVID-19 patients might benefit from the use of MP in its use in the long-term, with less pulmonary restrictive functions, attributed to fibrosis.

**Trial Registration:** ClinicalTrials.gov, Identifier: NCT04343729.

## Introduction

Most coronavirus disease 2019 (COVID-19) cases are either asymptomatic or of mild clinical presentation; nevertheless, a substantial number of individuals develop respiratory illness and require hospital care (1, 2). The most common clinical presentation of severe COVID-19 is acute respiratory failure consistent with acute respiratory distress syndrome (ARDS) and multiorgan failure (2). SARS-CoV-2 targets the receptor angiotensin-converting enzyme 2 (ACE-2) in human cells and causes the activation of alveolar macrophages and subsequent inflammatory response (3). This further induces airway inflammation, impairs alveolar gas diffusion, reduces lung ventilation, and worsens ventilation–perfusion mismatch (4, 5). An abrupt increase in inflammatory mediators can explain lung tissue damage in patients with COVID-19, known as “cytokine storm” (6). The release of proinflammatory cytokines increases the risk for vascular hyperpermeability, abnormal blood clotting, and multiple organ damage (7). The use of corticosteroids may help control this cytokine storm, mitigating tissue damage and ultimately fibrosis (8).

Despite robust evidence on the improvement of ARDS secondary to glucocorticoids (9), their efficacy on respiratory distress and ARDS secondary to viral infections have been controversial for influenza, SARS-CoV-1, and MERS-CoV (10–13), and COVID-19 is not an exception (14–17). However, until now, the impact of glucocorticoids in the long-term complications of COVID-19 has not been totally elucidated as most of the trials have mortality short-term primary endpoints (16–18), lacking a prospective cohort study design able to better understand this association. The longest known follow-up period to date was 60 days in a study in which intravenous dexamethasone at 24 h of moderate-to-severe ARDS onset has shown to significantly reduce all-cause mortality (19). The long-term complications of COVID-19 are still being published as the major cohorts are being followed, and their relevance is still a worldwide matter of debate (20). The aim of this study was to evaluate the postacute effects of corticosteroids used during the acute phase of severe COVID-19 in hospitalized patients and determine whether a short course of MP is associated with improvement in respiratory function at day 120 (D120) follow-up visit in these patients.

## Materials and Methods

### Study Design

Metcovid was a parallel, double-blind, randomized, and a placebo-controlled phase IIb clinical trial carried out between April 18 and October 9, 2020. The study aimed to assess the safety and efficacy of intravenous methylprednisolone (MP) sodium succinate (0.5 mg/kg) two times daily for 5 days in hospitalized patients with suspected COVID-19. Methods, eligibility criteria, and data on the major results until day 28 (D28) (primary outcomes) have already been published elsewhere (15). We followed-up all survivors and performed pulmonary function and physical capacity tests at D120 posthospital discharge. These data are presented here.

### Study Location and Population

Metcovid trial was conducted at *Hospital e Pronto-Socorro Delphina Rinaldi Abdel Aziz*, in Manaus, Western Brazilian Amazon (the largest public reference unit with a 100-intensive-care-bed capacity dedicated exclusively for the treatment of severe COVID-19 cases in town). Metcovid trial included hospitalized patients with clinical–radiological suspicion of COVID-19, aged 18 years or older at the time of inclusion, with oxygen saturation (SpO_2_) ≤ 94% in room air or requiring supplementary oxygen or under invasive mechanical ventilation (IMV). All discharged participants were asked to return for a follow-up visit on D120 after study randomization. All participants who died between D28 to D120 and who refused to return to the hospital were excluded, as were patients with altered blood pressure levels, which consists of a contraindication to perform spirometry tests, and poor-quality spirometry, which invalidates the test.

### Procedures

#### Procedures on Enrollment (Metcovid Trial: D1)

Computed tomography (CT) imaging, clinical, and laboratory information throughout hospitalization (from the day of enrollment to D28) are also presented for all surviving patients. Briefly, hematology and biochemistry analyses were performed in automated machines. Plasma samples from D1 were used for the measurement of human IL-6 by enzyme-linked immunoassay (ELISA) following the manufacturer's recommendations (R&D Systems, DY206). Two nasopharyngeal or one oropharyngeal swab (per institutional protocol) were used to extract viral ribonucleic acid (RNA) and were tested for SARS-CoV-2 using the one-step multiplex reverse transcription quantitative real-time polymerase chain reaction (RT-qPCR). Randomization procedures have already been published elsewhere (15). Briefly, an independent statistician prepared an electronically generated randomization list with 14 blocks of 30 participants per block, generated *via* R software version 3.6.1 (Blockrand package), accessible only to the study non-blinded pharmacists. Participants were randomized by the study pharmacist at the time of inclusion and were subsequently identified by their participant identification number.

#### Procedures on Long-Term Follow-Up (D120)

The D120 follow-up visit consisted of applying several instruments to evaluate the participant's respiratory functional status, including pulmonary function test, maximal respiratory pressures testing (manovacuometry), Duke activity status index (DASI) questionnaire, 6-min walk test, vital signs, and symptoms assessment. Need for medications between D28 and D120 was also registered. All data were recorded online in an electronic medical recording system (Medview version 710801 and Esthor) and then registered in an electronic database (REDCap).

### Pulmonary Function and Maximal Respiratory Pressures Testing

The pulmonary function test was assessed using the Microquark spirometer (Cosmed^®^, Italy). The test was carried out following recommendations of the American Thoracic Society/European Respiratory Society (ATS/ERS) guidelines (21). Briefly, the following parameters were assessed: forced vital capacity (FVC), forced expiratory capacity at the first second of exhalation (FEV1), FEV1/FVC ratio, forced expiratory flow (FEF 25-75), peak expiratory flow (PEF) according to their predicted values (21). All participants were given eight consecutive opportunities at max to perform two acceptable curves in the pulmonary function test (21). Only participants who performed maneuvers graded A and B, which are considered as acceptable curves according to the manufacturer's recommendations, were included and analyzed. Participants presenting to the D120 follow-up visit with altered blood pressure levels or incapability of following test instructions did not perform the test. All pulmonary function test measurements were performed by three trained and harmonized study staff.

Maximal respiratory pressure assessment consists of a non-invasive method for evaluating respiratory muscle strength (RMS). The test was performed using the MVD300-U digital manovacuometer (Homed^®^, Brazil). Testing was performed according to the ATS/ESR recommendations (22). Maximum inspiratory pressure (PI_max_) and maximum expiratory pressure (PE_max_) were evaluated.

### Other Functional Assessments

The Duke activity status index (DASI) is a standardized questionnaire correlated with gold-standard measures of functional capacity (23). It has been developed and validated against exercise test derived metabolic equivalents and peak oxygen consumption and consists of a 12-item self-reported questionnaire about usual physical activities. The minimum possible DASI score is 0, and the maximum possible score is 58.2. Higher scores indicate greater levels of fitness.

The 6-min walk test (6MWT) is a submaximal exercise test that evaluates functional capacity. The test captures concomitant extrapulmonary manifestations of respiratory, cardiovascular, frailty, muscle alterations, and others. It consists of the participant walking at their own pace in a 30-meter unobstructed long corridor for 6 min. The walked distance (6MWD) is then measured in meters. Before and after the test, heart rate, blood pressure, peripheral oxygen saturation (SpO_2_), and Borg dyspnea sensation were measured. The assessment was performed according to ATS guidelines (24). To show the real physical capacity we used the percentage of the predicted value for the Brazilian population (25).

### Statistical Analysis

The sample size calculation for the primary endpoints of the Metcovid trial is described elsewhere (15). Briefly, sample size was calculated assuming a 50% case-lethality rate among critically ill patients (26) and that MP would reduce case-lethality by 50%. Accounting for a test of differences in proportions between the two groups of the same size, a power of 80% and an alpha of 5%, a total of 378 participants were needed (189 per group). With the addition of 10% for losses, a final sample size of 416 participants was obtained. Sampling was performed with R software version 3.6.1 (TrialSize and gsDesign packages).

The D120 follow-up visit was performed only in patients who have survived and agreed to perform this visit. Pulmonary function outcomes were exploratory in the Metcovid trial, and therefore, the sample size calculation was not performed for these outcomes. To ensure that the effect of randomization was not lost, univariate analysis on baseline variables was performed to check for differences among the groups. Therefore, a multivariate log-binomial generalized linear regression analysis was performed regarding pulmonary functional assessment to avoid eventual biases between the groups. Mean and standard deviation (SD) were calculated when distributions were confirmed as normal. Otherwise, data were expressed as medians and interquartile ranges (IQR). For qualitative variables, Chi-square or Fisher's exact test were used accordingly. *T*-test or Wilcoxon Ranksum test were used for means or median comparisons when appropriate. Furthermore, to analyze the effects size of functional and respiratory measurements, we used the G^*^Power 3.0 software. Cohen's *d* for interpretation was accepted. A significance level lower than 5% was considered. Stata 13.0 software was used.

### Ethical Aspects

This study was conducted following the Declaration of Helsinki principles and the Good Clinical Practice guidelines of the International Conference on Harmonization. The protocol was approved by the Brazilian Committee of Ethics in Human Research (CONEP – CAAE 30615920.2.0000.0005), clinical Trials registration number NCT04343729. All hospitalized patients were screened for study eligibility criteria. Eligible participants were informed about the objectives, risks of participation, and only then were invited to participate. All were given time to carefully read and then sign an informed consent form (ICF). If the participant was not able to properly consent study participation, e.g., sedated at the ICU, the consenting process was performed with the participant's legal representatives. For study participants who could not sign themselves the ICF during the hospitalization period, a reconsent was performed at the D120 follow-up visit.

## Results

**Figure 1:**
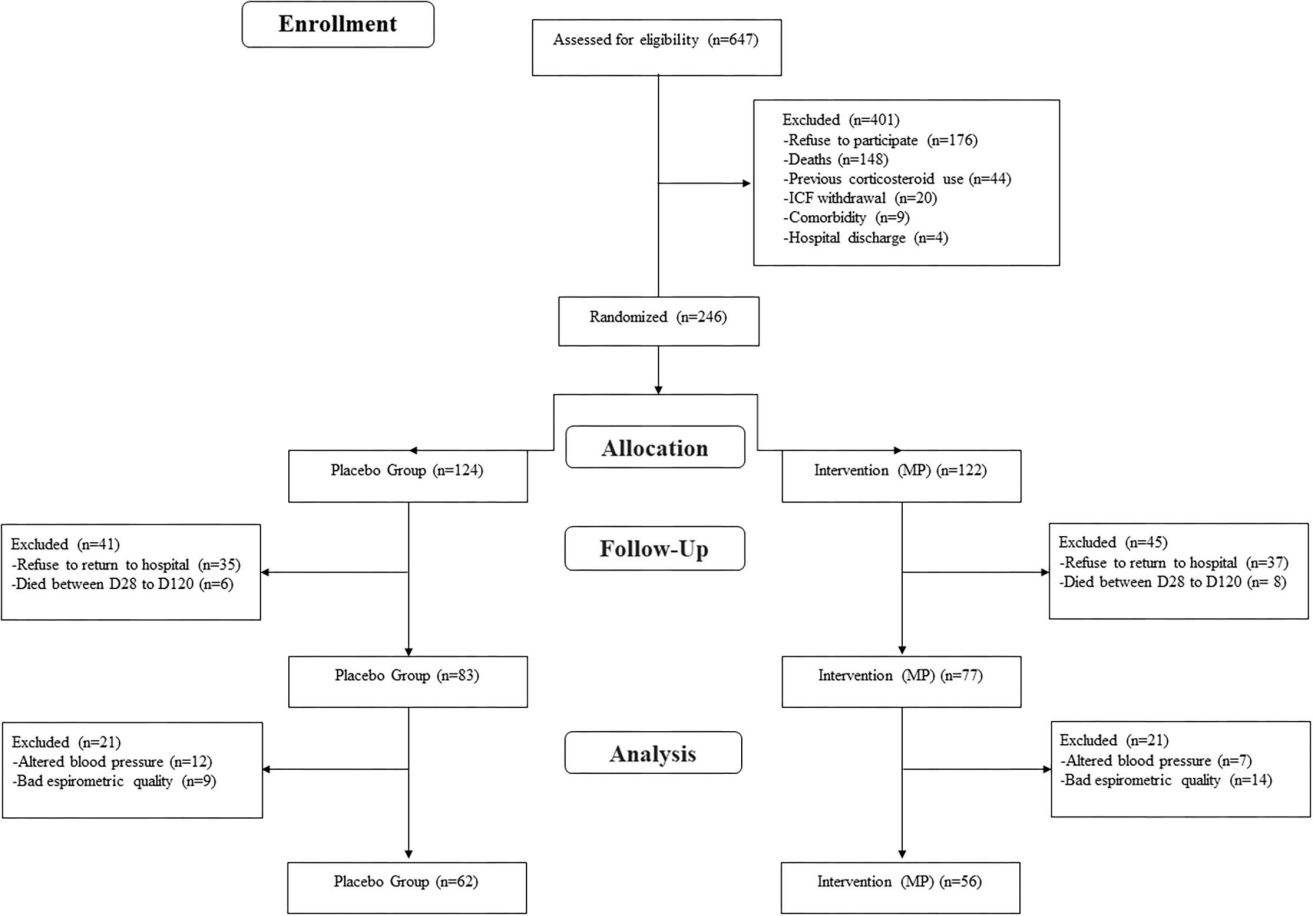
Study flow chart of the Metcovid trial survivors who performed pulmonary function test.

A total of 246 patients completed the D28 follow-up in the Metcovid trial. The details and results of the trial are presented elsewhere (15). Out of these, a total of 14 (5.7%) patients died between D28 and D120 follow-up visits, and 72 (29.3%) refused to come back for additional evaluation. Importantly, Manaus was still presenting a high number of cases and deaths due to COVID-19 by the time of this study and there was no vaccine available, which may account for the high rate of refusals. A total of 160 (65.0%) were, therefore, evaluated on day 120 follow-up visit, of which 118 (73.75%) underwent satisfactory pulmonary function testing (62 in the placebo arm and 56 in the MP arm) (Figure 1). The data of these participants are presented here.

Baseline characteristics (D1, at hospital admission) of the 118 analyzed patients are shown in Table 1. Of these, most patients were men (*n* = 64, 54.2%), of admixed race (*n* = 86, 72.9%) with a mean age of 49.3 ± 13.2 years and a median body mass index of 30.2 kg/m^2^ (IQR: 27.0–35.4). Age was not different at baseline between groups 49.3 (14.7) and 49.3 (11.5) (*P* = 0.06) for the control and MP groups, respectively. The SARS-CoV-2 RT-PCR was positive for 91 (86.7%) participants (47 [87.0%] and 44 [86.3%] in placebo and MP, respectively). The distribution of comorbidities (i.e., diabetes, chronic obstructive pulmonary disease, and hypertension) or even smoking history was similar between groups. Groups were homogenous regarding all previous variables. Nonetheless, and despite following all eligibility criteria and blinded randomization procedures at hospital admission, but considering that only those who survived and agreed to perform the day 120 follow-up visit, the baseline IL-6 levels were higher in the MP group (*P* < 0.01). Also, the distribution of participants presenting major clinical outcomes, as the need for ICU on admission (*P* = 0.06) and IMV (*P* = 0.07), tended to be different between groups, but not statistically significant. All baseline (D1) demographic, clinical, laboratory, and radiological findings of survivors of COVID-19 which returned on follow-up visit at D120 follow-up visit, and major therapeutical interventions from D1 to D120, are presented. No major differences were seen.

**Table 1:** Baseline (D1) demographic, clinical, laboratory, and radiological findings of survivors of COVID-19 which returned on follow-up visit 120 days, and major therapeutical interventions from D1 to D120.

	**Total *n =* 118**	**Placebo *n =* 62**	**MP *n =* 56**	***P***
Age, years, mean (SD)	49.3 (13.2)	49.3 (14.7)	49.3 (11.5)	0.06
Gender (women) *n* (%)	54 (45.8)	31 (50.0)	23 (41.1)	0.33
BMI, kg/m^2^, median (IQR)	30.2 (27.0–35.4)	31.9 (27.5–35.6)	29.6 (26.3–34.3)	0.23
Smoking history *n* (%)	47 (39.8)	21 (33.9)	26 (46.4)	0.16
Alcohol use disorder *n* (%)	33 (28.0)	20 (32.3)	13 (23.2)	0.27
Diabetes *n* (%)	30 (25.4)	17 (27.4)	13 (23.2)	0.60
Chronic pulmonary obstructive disease, *n* (%)	1 (0.9)	1 (1.6)	0 (0.0)	1
Hypertension *n* (%)	43 (36.4)	23 (37.1)	20 (35.7)	0.88
ICU on admission *n* (%)	6/94 (6.4)	1/50 (2.0)	5/44 (11.4)	0.06
IMV on admission *n* (%)	6 (5.1)	1 (1.6)	5 (8.9)	0.07
Oxygen saturation (SpO_2_) median (IQR)	97.0 (95.0–98.0)	97.0 (96.0–98.0)	96.0 (95.0–98.0)	0.17
Creatine kinase, U/L, median (IQR)	76.3 (39.9–144.1)	60.3 (32.4–120.3)	87.4 (46.3–194.1)	0.05
Creatine kinase MB, U/L, median (IQR)	18.2 (12.0–23.1)	18.3 (12.8–20.7)	16.3 (10.9–24.0)	0.90
Lactate dehydrogenase, U/L, median (IQR)	617.0 (307.0–892.0)	473.0 (307.0–845.0)	681.5 (267.0–942.0)	0.57
D–dimer, ng/mL, median (IQR)	641.3 (458.0–1576.2)	830.0 (471.1–1576.2)	511.4 (451.6–704.0)	0.27
C–reactive protein, mg/L, median (IQR)	66.7 (29.1–87.3)	57.4 (15.5–83.5)	73.3 (45.8–90.8)	0.10
IL−6, pg/mL, median (IQR)	31.8 (8.3–82.2)	15.7 (6.8–61.9)	52.6 (17.4–131.0)	**<0.01**
Ground-glass opacity infiltration n/N (%)	84/89 (94.4)	43/46 (93.5)	41/43 (95.3)	0.70
Consolidation n/N (%)	79/89 (88.8)	40/46 (87.0)	39/43 (90.7)	0.58
Unilateral consolidation n/N (%)	7/89 (7.9)	4/46 (8.7)	3/43 (7.0)	0.76
Bilateral consolidation n/N (%)	72/89 (80.9)	36/46 (78.3)	36/43 (83.7)	0.51
Pleural effusion n/N (%)	18/89 (20.2)	7/46 (15.2)	11/43 (25.6)	0.22
qSOFA score ≥2 *n* (%)	16 (13.6)	8 (12.9)	8 (14.3)	0.83

*MP, methylprednisolone; SD, standard deviation; ICU, intensive care unit; IMV, invasive mechanical ventilation; BMI, body mass index; kg, kilogram; m, meter; IQR, interquartile range; IL, interleukin; qSOFA, quick Sepsis Related Organ Failure Assessment. Bold value means statistically significant p-values*.

Regarding the pulmonary function testing, the FEV1 and FVC values were statistically higher in the MP group at D120 follow-up visit, showing better outcomes in this group. Table 2 shows the results of pulmonary function tests and RMS among the studied population in the follow-up visit. In the univariate analysis, FEV1 (2.6, [0.7], *P* = 0.01) and FVC (3.2, [0.8], *P* = 0.01) are significantly higher in patients in the MP arm. Furthermore, both variables showed moderate effects size (0.40 and 0.37, respectively). In the multivariate analyses, controlled for IMV and IL-6 levels in baseline, FVC (3.2, [0.8], *P* = 0.01) still showed to be significantly higher in MP arm. The percent of predicted FEV1 and FVC in the total number of patients were within normal range (87.0% ± 21.5 and 94.6% ± 39.5, respectively), with no significant differences between the groups (*P* = 0.20). There was no statistically significant difference between groups for the other pulmonary function parameters.

**Table 2:** Functional and respiratory measurements outcomes on D120 follow-up visit.

	**Total *n* = 118**	**Placebo *n* = 62**	**MP *n* = 56**	**Univariate *P***	Multivariate^*^ *P*	**Effects size *Cohen's D***
FEV_1_ percent of predicted (%, SD)	87.0 (21.5)	84.6 (25.4)	89.7 (15.9)	0.20	0.40	0.24
FEV_1_ (L, SD)	2.4 (0.8)	2.3 (0.8)	2.6 (0.7)	**0.01**	0.12	0.40
FVC percent of predicted (%, SD)	94.7 (39.5)	96.9 (53.0)	92.3 (13.4)	0.52		0.11
FVC (L, SD)	3.0 (0.8)	2.9 (0.8)	3.2 (0.8)	**0.01**	**0.01**	0.37
FEV_1_/FVC	79.4 (11.7)	78.4 (14.0)	80.5 (8.4)	0.33	0.94	0.18
PImax (cm H_2_O, SD)	93.9 (32.3)	96.0 (34.6)	91.8 (29.9)	0.49	0.74	0.13
PEmax (cm H_2_O, SD)	107.8 (35.5)	107.8 (36.6)	107.7 (34.7)	0.99	0.18	**<0.01**
DASI *V*O*_2_ peak* estimate(mL/kg/min, SD)	27.6 (6.0)	27.1 (6.0)	28.2 (6.1)	0.31	0.11	0.18
DASI score, SD	41.9 (14.1)	40.6 (13.9)	43.3 (14.2)	0.31	0.10	0.19
6MWD (m, SD)	385.9 (89.4)	384.2 (93.8)	387.7 (85.4)	0.84	0.23	0.03
6MWD percent of predicted (%, SD)	64.9 (19.8)	63.0 (22.1)	66.9 (17.0)	0.29		0.19

*FVC, Forced vital capacity; FEV1, Forced expiratory volume on 1^st^ second; PEF, peak expiratory flow; PImax, Maximum static inspiratory pressures; PEmax, Maximum static expiratory pressures; 6MWD, 6 min walk distance; DASI, Duke Activity Status Index; VO_2_, oxygen uptake; SD, standard deviation; IQR, interquartile range*.

* *Controlled for IMV need and IL-6 levels. Bold value means statistically significant p-values*.

## Discussion

This study evidences that a short course of intravenous MP during the acute phase of COVID-19 in hospitalized patients resulted in significant and statistically higher FEV1 and FVC respiratory functional parameters at a D120 follow-up visit, showing a positive lasting effect of the MP intervention for this population in comparison with the other group. Corticosteroids seem to play an important role in reducing lung tissue damage during the unregulated inflammatory process, a prominent feature of ARDS associated with SARS-CoV-2 infection (8, 27, 28). Also, the use of corticosteroids in COVID-19 is supported by the existing evidence of randomized clinical trials and observation studies; nonetheless, the type, dosage, starting time, and duration still need more research (14, 29, 30). Despite that, the latest guidelines recommend using a short course of systemic corticosteroids over not using corticosteroids in adults with severe or critical COVID-19 (31). The long-term pulmonary sequelae of COVID-19 are still unknown. Viral lung infections by SARS-CoV-1, MERS, and influenza H1N1 and H7N9 reveal that after recovery, patients may suffer irreversible pulmonary dysfunction and present residual images or functional abnormalities, which can also occur in COVID-19 (32–40). In this study, participants who were randomized in the MP group had improved respiratory function within a 120-day period, which seems to be a relevant effect of the use of corticosteroids in the long-term for severe and critical COVID-19 patients, which gives new venues regarding the benefits of such therapy in the long-term.

Evidence of the pulmonary function profile in patients with COVID-19 is currently limited to reasonably small sample studies and short-term follow-up, especially in critical participants. However, patients may present improvement in respiratory function and quality of life about 6 weeks after the disease, but there are still some degrees of restrictive abnormalities (41). Studies show discrete changes in pulmonary function parameters (changes in FVC, FEV1, and FEV1/FVC parameters) with restrictive changes, and to a lesser extent obstructive disorders at the time of hospital discharge and 30 days postinfection (42–47), an aspect also present in this study 120 days after beginning the treatment, with patients who used MP presenting less impairment.

In our study, FEV1 and FVC of patients in the MP group were higher than in the control group. In the multivariate analysis, FVC persisted significantly higher, independently of IMV need and IL-6 levels (a proxy of level of inflammation during the acute phase). IL-6 was higher in MP arm in the baseline, indicating that the MP group had more inflammation at baseline, and still, both FEV1 and FVC were higher in the study intervention group, which may indicate that the intravenous MP during hospitalization may help in long lung protection. Wang JY and colleagues (46) did not observe changes in predicted FEV_1_ (81.1%) and FVC (82.5%); moreover, slight impairment of the diffusion capacity corrected for carbon monoxide (DLCOc, 67.08 of the predicted value) after discharge was visualized. Otherwise, Zhao and colleagues (32) demonstrated a low frequency of spirometry changes (CPT reduction in 7.27, FEV_1_ in 10.91, FVC in 10.91, DLCO in 16.36, and alteration of small airways in 12.73) and a positive correlation between D-dimer levels at admission and diffusion deficiency (27). In our study, we did not evaluate the gas diffusion in the lung (DLCo).

Several autopsy studies showed histological alterations consistent with ARDS in the lungs of COVID-19 patients (48–51). Pulmonary fibrosis is a very-well recognized consequence of ARDS. A fibrotic pattern has been shown to occur in up to 22% of deceased patients with COVID-19, generally 3 weeks after symptoms onset, and mainly in those with preexisting lung diseases (46). For instance, corticosteroids have presented efficacy in treating acute fibrinous and organizing pneumonia, an acute diffuse lung injury (52).

Despite evidence of early stage pulmonary tissue abnormalities in COVID-19-associated ARDS, especially when comparing survivors and non-survivors (53), the persistence of pulmonary sequelae in recovering COVID-19 patients has been a matter of increasing debate (8). Evidence from literature shows that more than 70% of participant who recover from ARDS, irrespective of the underlying cause, develop some sort of abnormal imaging after 6 months (54). Nonetheless, preliminary evidence on patients with mild-to-moderate forms of COVID-19 has shown recovery over-time of pulmonary opacities in CT imaging, with no evidence of pulmonary fibrosis (20). On the other hand, high resolution CT performed in SARS-CoV-2-related ARDS survivors treated with prolonged low-dose MP after hospital discharge demonstrated parenchymal alterations at 3 months after discharge from severe COVID-19 pneumonia (55). The present study shows that FEV1 and FVC were within the normal range, but increased in the MP group. However, although both groups had similar CT impairment in baseline, the MP arm developed less respiratory dysfunction, which could indicate resolution and/or functional adaptation to exercise. This needs to be properly investigated in the future. The results of this study may shed light on the benefit of a short-course of intravenous MP early in hospitalization, leading to pulmonary protection, in some extent, even after 120 days.

This study has several limitations. Lung function testing was not performed during hospitalization or discharge. This compromised the comparison of respiratory functional changes between the acute disease and at discharge with the postacute phase. Also, the DLCo was not measured, which could shed further insights into alveolar tissue damage and its repercussion on functional capacity. Nonetheless, it is important to mention that spirometry is widely available and used more routinely, becoming more applicable in clinical practice, especially in low and middle incomes countries, in comparison to assessing the diffusing capacity of the lungs for carbon dioxide. Many hospitalized participants in the Metcovid trial were unwilling to return to the D120 follow-up visit due to several reasons, which further reduced the final study sample. Also, not all were able to perform adequate respiratory functional testing. However, we have mitigated this effect by performing regression analyses.

In conclusion, a short course of intravenous MP was found to be associated to better respiratory functional assessments 120 days after hospitalization when used in the acute phase of COVID-19 in hospitalized patients. These results seem to indicate that this intervention could have an important role in long-term pulmonary protection for this type of patients. It is important to mention that intravenous MP was the intervention studied in the Metcovid trial, and results from respiratory functional assessments performed 120 days after hospitalization, which are described here, are probably results from the combination of other concomitant therapies and clinical management during hospital care, which were equally available to all participants in both the groups. Larger sample sizes and more extended follow-up periods are still necessary to better understand chronic outcomes of severe and critical COVID-19 clinical presentations.

## Group Information

**Metcovid Team (in alphabetical order):** Adria de Lemos Vasconcelos, Adriana Ferreira Praia Marins, Alexandre de Oliveira Trindade, Aline Sales Mendes Záu, Amanda Carvalho de Oliveira, Ana Carolina Azevedo Furtado, Ana Paula Coelho Rocha, Anderson da Silva Souza, Andiana de Souza Dias, Aníbal Belém, Antonny Michael da Silva Sousa, Beatriz França da Silva, Beatriz Leitão Franco, Bleno Leonam Gonçalves da Costa, Carla C. Judice, Carlos Eduardo Padron de Morais, Danielle Severino Sena da Silva, Debora Camila Gomes Duarte, Ejandre Garcia Negreiros da Silva, Elias da Silva Lemos, Elizandra Freitas do Nascimento, Elson Silva de Almeida, Elyana Almeida Marques, Emanuel Medeiros Marinho de Almeida, Emanuelle Lira da Silva, Ester Galvão dos Santos, Ezequiel da Silva Oliveira, Fábio Manabu Martins Shimizu, Fabíola Ramalho Ferreira de Souza, Felipe da Silva do Vale, Fernanda dos Santos de Almeida Lima, Fernando Hugo Jesus da Fonseca, Flávia Alencar Fontenelle, Francielen de Azevedo Furtado, Gabrielle Da Silva Pereira, Geísa Aleixo Bezerra, Guilherme Kemeron Maciel Salazar, Handerson da Silva Pereira, Hilda Ferreira de Melo, Ingrid Nascimento Oliveira, Ivanildo Vieira Pereira Filho, Jaily e Silva Rosa, Jacimara Vasques Gomes, Jonas Mota Lemos, Josué Nélio Brutus, Karina Pinheiro Pessoa, Laleyska Deucylane Costa Rodrigues, Larissa Esthefani Barros Cirino, Lauro Fragata Mourão Filho, Leandro Moura, Lorenna Pereira de Souza, Lucas Barbosa Oliveira, Marcela Menezes dos Santos, Marcus Vinicius Ramos da Silva, Mauro Pereira Rodrigues, Mayara Tavares de Menezes, Micaela Maciel dos Santos Mota, Nadya Fonseca Corrêa, Najara Bittencourt, Nagila Morais Rocha, Natália Guedes de Melo Silva, Priscilla de Oliveira Saraiva, Quézia de Sousa Monteiro, Rafael Theodoro dos Santos, Rebecca Augusta de Araújo Pinto, Rodrigo Saboia de Lima Reinan Brotas Ferreira, Rosângela Francisca Tanantas de Melo, Sabrina Teixeira Saenz, Salete Sara Alvarez Fernandes, Sheila Vítor-Silva, Thalie Cavalcante Santos, Tânia Maria Rodrigues de Oliveira, Tatyana A. Tavella, Thais Tavares Câmara, Thiago Serrão Pinto, Tilza Waleska Rocha dos Santos, Valdinete Alves do Nascimento, Wanessa Pessoa Sousa Barbosa, Wellinthon Ferreira de Melo, Wlademir Braga Salgado Sobrinho.

## Data Availability Statement

The original contributions presented in the study are included in the article/Supplementary Material. The raw data supporting the conclusions of this article will be made available upon request to the corresponding author.

## Ethics Statement

The studies involving human participants were reviewed and approved by Brazilian Committee of Ethics in Human Research. The patients/participants provided their written informed consent to participate in this study.

## Author Contributions

CB, RF, GM, MS, FV, VS, MX, LH, WM, and ML: concept and design. CB, VS, MA-A, DB, LH, FG, MX, MR, RF, EF, AR, MF, JB-S, FV, WM, and ML: acquisition, analysis, or interpretation of data. CB, DB, VS, FV, WM, and ML: drafting of the manuscript. CB, MS, FV, VS, MA-A, DB, JB-S, MX, LH, FG, FC, WM, and ML: critical revision of the manuscript for important intellectual content. VS, MR, MX, BS, FV, and GA: statistical analysis. WM and ML: obtained funding. CB, JB-S, FG, GM, and RN: administrative, technical, or material support. CB, MS, FV, GM, MA-A, DB, LH, MX, WM, and ML: supervision.

## Funding

This study was funded by the Government of the Amazonas State, Coordenação de Aperfeiçoamento de Pessoal de Nível Superior (CAPES), Departamento de Ciência e Tecnologia/Ministério da Saúde (DECIT), Ministério da Ciência, Tecnologia e Inovações (MCTI), Conselho Nacional de Desenvolvimento Científico e Tecnológico (CNPq, Grant 403253/2020-9), Fundação de Amparo à Pesquisa do Estado do Amazonas (FAPEAM) (RESOLUÇÃO N. 008/2021 - POSGRAD 2021; EDITAL 006/2020) and PRO-ESTADO. The funder of the study had no role in study design, data collection, data analysis, data interpretation, or writing of the report. The corresponding author had full access to all data in the study and had final responsibility for submitting it for publication.

## Conflict of Interest

The authors declare that the research was conducted in the absence of any commercial or financial relationships that could be construed as a potential conflict of interest.

## Publisher's Note

All claims expressed in this article are solely those of the authors and do not necessarily represent those of their affiliated organizations, or those of the publisher, the editors and the reviewers. Any product that may be evaluated in this article, or claim that may be made by its manufacturer, is not guaranteed or endorsed by the publisher.
