# Supplementary material for: Short-Course of Methylprednisolone Improves Respiratory Functional Parameters After 120 Days in Hospitalized COVID-19 Patients (Metcovid Trial): A Randomized Clinical Trial
Source: Front Med (Lausanne). 2021 Nov 30;8:758405. doi: 10.3389/fmed.2021.758405 (PMC8669506; doi:10.3389/fmed.2021.758405)
Supplement: Supplementary file 1 [file Table_1.DOCX]

**Supplementary table 1.** Major therapeutic interventions from D1 to D120 and another baseline (D1) clinical and laboratory characteristics of survivors of COVID-19 which returned on follow-up visit 120 days,

|  | **Total**  **n=118** | **Placebo**  **n=62** | **MP**  **n=56** | ***P*** |
| --- | --- | --- | --- | --- |
|  |  |  |  |  |
| Positive blood culture n/N (%) | 1/73 (1.4) | 1/39 (2.6) | 0/34 (0.0) | .35 |
| White blood cell count, 10^3^/mm^3^, mean (SD) | 10.5 (5.6) | 9.9 (4.1) | 11.3 (6.9) | .18 |
| Hemoglobin, g/dL, mean (SD) | 12.5 (1.7) | 12.3 (1.7) | 12.7 (1.6) | .14 |
| Lymphocytes, mean (SD) | 15.9 (8.8) | 17.4 (8.6) | 14.2 (8.8) | .05 |
| Platelet count, 10^3^/mm^3^, mean (SD) | 307.4 (124.0) | 323.3 (123.6) | 289.8 (123.2) | .15 |
| Blood glucose, mg/dL, median (IQR) | 163.0 (128.0-209.0) | 161.5 (123.0-197.0) | 170.5 (136.0-223.0) | .39 |
| Alanine aminotransferase, U/L, median (IQR) | 56.1 (36.5-82.2) | 63.6 (34.9-87.4) | 51.1 (36.8-70.7) | .25 |
| Creatinine, mg/dL, median (IQR) | 0.9 (0.7-1.0) | 0.9 (0.7-1.0) | 0.9 (0.7-1.0) | .52 |
| Need for other steroids at clinical discretion n/N (%) | 31/115 (27.0) | 20/61 (32.8) | 11/54 (20.4) | .13 |
| Need for anticoagulants n/N (%) | 80/113 (70.8) | 39/59 (66.1) | 41/54 (75.9) | .25 |
| Need for bronchodilators n/N (%) | 30/115 (26.1) | 12/61 (19.7) | 18/54 (33.3) | .09 |
| Need for antibiotics n/N (%) | 98/115 (85.2) | 51/61 (83.6) | 47/54 (87.0) | .60 |
| Days from illness onset to randomization, median (IQR) | 13.0 (10.0-17.0) | 12.0 (9.0-16.0) | 14.0 (10.0-18.5) | .17 |
| Days from IMV to randomization, median (IQR) | 5.5 (5.0-8.0) | 6.0 (6.0-6.0) | 5.0 (5.0-8.0) | .77 |
| Time of IMV, days, Mean (SD) | 8.5 (4.5-14.5) | 5.0 (4.0-15.0) | 10.0 (6.0-14.0) | .64 |
| MP: methylprednisolone; SD: standard deviation; IMV: invasive mechanical ventilation; IQR:  interquartile range | | | | |
